# Essential Roles of Cyclin Y-Like 1 and Cyclin Y in Dividing Wnt-Responsive Mammary Stem/Progenitor Cells

**DOI:** 10.1371/journal.pgen.1006055

**Published:** 2016-05-20

**Authors:** Liyong Zeng, Cheguo Cai, Shan Li, Wenjuan Wang, Yaping Li, Jiangye Chen, Xueliang Zhu, Yi Arial Zeng

**Affiliations:** 1 State Key Laboratory of Cell Biology, CAS Center for Excellence in Molecular Cell Science, Institute of Biochemistry and Cell Biology, Shanghai Institutes for Biological Sciences, Chinese Academy of Sciences, Shanghai, China; 2 State Key Laboratory of Molecular Biology, Institute of Biochemistry and Cell Biology, Shanghai Institutes for Biological Sciences, Chinese Academy of Sciences, Shanghai, China; 3 School of Life Science and Technology, ShanghaiTech University, Shanghai, China; National Cancer Institute, UNITED STATES

## Abstract

Cyclin Y family can enhance Wnt/β-catenin signaling in mitosis. Their physiological roles in mammalian development are yet unknown. Here we show that Cyclin Y-like 1 (Ccnyl1) and Cyclin Y (Ccny) have overlapping function and are crucial for mouse embryonic development and mammary stem/progenitor cell functions. Double knockout of *Ccnys* results in embryonic lethality at E16.5. In pubertal development, mammary terminal end buds robustly express *Ccnyl1*. Depletion of Ccnys leads to reduction of Lrp6 phosphorylation, hampering β-catenin activities and abolishing mammary stem/progenitor cell expansion in vitro. In lineage tracing experiments, *Ccnys*-deficient mammary cells lose their competitiveness and cease to contribute to mammary development. In transplantation assays, Ccnys-deficient mammary cells fail to reconstitute, whereas constitutively active β-catenin restores their regeneration abilities. Together, our results demonstrate the physiological significance of Ccnys-mediated mitotic Wnt signaling in embryonic development and mammary stem/progenitor cells, and reveal insights in the molecular mechanisms orchestrating cell cycle progression and maintenance of stem cell properties.

## Author Summary

Stem cell self-renewal has two essential elements, cell division and at least of one of the daughter cells retaining stem cell properties, so-called stemness. The interconnections between cell cycle and cell fate specification have been explored in embryonic stem cells. However, less is known about how cell cycle affects the cell fate decision in tissue stem cells. In this study, we explore the function of particular mitotic factors Ccny and Ccnyl1 in regulating the dividing tissue stem cells. The development of the mammary gland occurs mostly in postnatal pubertal stage. At the time, the robustly dividing stem/progenitor cells reside at the forefront of the mammary epithelium extension, underlining the mammary gland as a good model to study the interconnection of cell cycle and tissue stem cells. In this study, we show that in dividing mammary stem/progenitor cells, Ccny and Ccnyl1 enhance Wnt signaling activities in mitosis. The signaling enhancement in this time window is essential for the stem/progenitor cell property maintenance during division. Deletion of Ccnys results in diminishing their competitiveness and developmental potential.

## Introduction

Stem cell self-renewal is tightly associated with cell cycle progression. In particular, active stem cells rapidly divide and coordinate with cell fate choices [1]. Active stem cells can be replenished by quiescent stem cells over time or upon injury [2]. Distinct from other somatic cell proliferation, the process of self-renewal needs to ensure stemness maintenance. The molecular mechanism overseeing stemness maintenance during division is poorly understood.

Wnt/β-catenin signaling plays a prominent role in adult stem cell self-renewal in many tissues [3]. The level of Wnt/β-catenin signaling in stem cells is meticulously regulated, while different activation levels of the signaling result in distinct fate decisions by stem cells [4–6]. Wnt signaling is initiated upon the binding of Wnt ligands to Frizzled and lipoprotein receptor-related proteins 5 and 6 (LRP5/6) receptors. Consequent abolishing or titrating away of the APC/Axin/GSK3 complex leads to β-catenin accumulation and translocation to the nucleus where it binds to Tcf/Lef family members, activating target gene expression [3]. A key step for stoichiometric regulation of Wnt signaling happens at the membrane, where Lrp6 receptor activation occurs in sequential steps before reaching its full competence. It is well established that Lrp6 is phosphorylated on PPPSP motif by GSK3 and on CK1 site by CK1γ [7,8]. Recently, an additional phosphorylation event of Lrp6 has been identified to precede Wnt ligand stimulation [9]. During mitosis, cyclin Y (Ccny) localized at the plasma membrane recruits Cyclin-dependent kinase 14 (Cdk14) for the phosphorylation of Lrp6 on PPPSP motif, which sensitizes Lrp6 for upcoming Wnt signals [9,10]. The finding of Lrp6 phosphorylation by Ccny/Cdk14 reveals a cell cycle dependent Wnt signaling activation mechanism, adding a new level of complexity to stoichiometric Wnt signaling activation [9,11]. Although enhancing the Wnt-receptor Lrp6 competence by Ccny is important for Xenopus development [9], whether a similar Ccny/Lrp6 regulatory event is physiologically significant in mammals and in stem cell biology is unknown.

The mammary gland is a bi-layered epithelial organ consisting of an inner layer of luminal cells and an outer layer of basal cells (myoepithelial cells). Mammary stem/progenitor cells are Wnt-responsive cells resided in the basal layer [12–14]. The mammary gland develops mostly in the postnatal stage. At the onset of puberty, at around 3 weeks of age in mouse, a rapid expansion of the rudimentary ductal tree begins. Branching ductal morphogenesis proceeds across the entire mammary fat pad, and is completed at approximately 7 weeks [15,16]. During puberty, at the growing tips of the ducts are the highly proliferative terminal end buds (TEBs), which are believed to house active mammary stem cells (or transient amplifying cells), actively cycling to fuel the growth spurt [17,18]. This notion is further supported by a lineage tracing study in which labeled TEB cells undergo massive clonal expansion, giving rise to ample progeny cells in development [13].

In this study, we applied a genetic approach to investigate the roles of Ccny and its paralogue Ccnyl1 in active stem/progenitor cells and to examine their functions in mammary development and regeneration. Our study establishes that mitosis-induced and Ccny family-mediated Wnt signaling activity is essential for keeping the developmental potential of dividing mammary stem/progenitor cells, shedding light in the molecular mechanisms coordinating cell cycle progression and undifferentiated state maintenance.

## Results

### *Ccny* and *Ccnyl1* have overlapping roles in development

**Fig 1:**
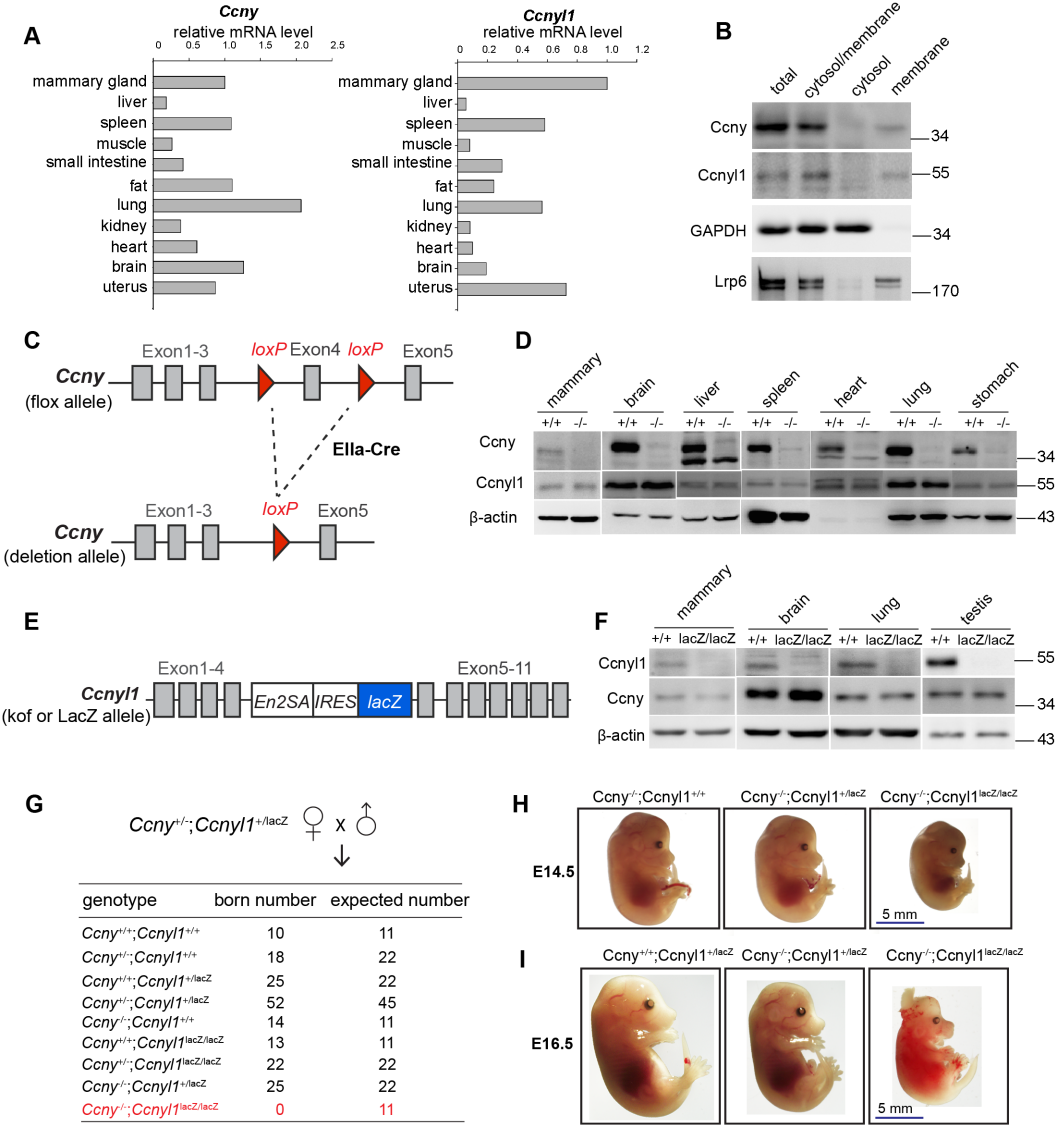
Generation of *Ccny* and *Ccnyl1* mutant mice. **(A)** qPCR analysis of mouse *Ccny* and *Ccnyl1* mRNA levels in different tissues isolated from a 6-week-old CD1 mouse. **(B)** Membrane localization of Ccnys. Mouse mammary epithelial EpH4 cells were fractionated into cytosol and membrane fractions. GAPDH and Lrp6 serve as cytosol and membrane loading control, respectively. **(C)** *Ccny* gene targeting strategy. Exon 4 was flanked by two loxP sites. *Ccny*^*flox/+*^ mice were crossed with EIIa-Cre, which can induce recombination in germ cells and transmit the genetic alteration to progeny *Ccny*^*+/-*^ mouse. **(D)** Western analysis to confirm the whole-body knockout of Ccny in *Ccny*^*-/-*^ mouse. Lysates of different organs were prepared from a 12-week-old *Ccny*^*-/*-^ mouse and its littermate wildtype control mouse. The lysates were analyzed by western with anti-Ccny and anti-Ccnyl1 antibody. β-actin served as loading control. **(E)** *Ccnyl1*^*lacZ/+*^ reporter mice gene targeting strategy. **(F)** Western analysis to confirm the knockout efficiency of Ccnyl1 in *Ccnyl1*^*lacZ/lacZ*^ mouse. Lysates of mammary gland, brain and lung were prepared from a 5-week-old female *Ccnyl1*^*lacZ/lacZ*^ mouse and its wildtype female littermate. Testis lysates were prepared from an 8-week-old male *Ccnyl1*^*lacZ/lacZ*^ mouse and its wildtype male mice. The lysates were analyzed by western with anti-Ccnyl1 and anti-Ccny antibodies. β-actin served as loading control. **(G)** No double knockout mice (*Ccny*^*-/-*^;*Ccnyl1*^*lacZ/lacZ*^) were born from indicated cross, while the other genotypes displayed the expected Mendelian ratios. **(H)** Representative images of a *Ccny*^*-/-*^;*Ccnyl1*^*lacZ/lacZ*^ embryo and its control littermates at E14.5. The double knockout embryo has smaller body size (n = 3 embryos per group). **(I)** Embryonic lethality of the double knockout embryo at E16.5 (n = 3 embryos per group).

We first investigated the expression patterns of Ccny and Ccnyl1, hereafter referred to collectively as Ccnys. We found that both Ccnys, which share high similarity in amino acid sequence (S1A Fig), are expressed in many tissues, including the mammary gland (Fig 1A). We generated Ccny and Ccnyl1 polyclonal antibodies and validated their specificity (S1B–S1D Fig). Cell fractionation and Western analyses indicated membrane localization of Ccnyl1, similar to that of Ccny (Fig 1B) [10].

To investigate the function of Ccny, we generated *Ccny* conditional mutant mice, with two loxP sites inserted to flank exon 4 (Fig 1C and see Methods for details). To create *Ccny*^+/-^ mutant mice, male *Ccny*^flox/+^ mice were bred with female EIIa-cre mice, inducing recombination in germ cells and transmitting the *Ccny* deletion to progeny. The resulting *Ccny*^+/-^ mice went through additional cross to generate *Ccny*^-/-^ mice (Fig 1C). We confirmed the deletion of Ccny in various tissues by western analyses, and the deletion of Ccny did not affect the level of Ccnyl1 (Fig 1D). The *Ccny*^-/-^ mice were grossly normal and their mammary glands displayed no discernable phenotypes (S2 Fig), suggesting a possible compensation by *Ccnyl1*. To address this, we utilized a *Ccnyl1* knock-in mouse line (*Ccnyl1*^*lacZ/+*^) generated in EUCOMM, in which a *LacZ* cassette was inserted into the intron between exon 4 and 5 (Fig 1E). Although the insertion disrupted the *Ccnyl1* transcription, *Ccnyl1*^lacZ/lacZ^ mice were viable and also exhibited normal mammary gland morphology (S2 Fig). The deletion of Ccnyl1 was also validated in multiple tissues while the level of Ccny was not affected (Fig 1F). Interestingly, the *Ccnys* double knockout mice (*Ccny*^-/-^;*Ccnyl1*^*lacZ/lacZ*^; DKO) were embryonic lethal (Fig 1G). At E14.5, *Ccnys* DKO embryos appeared smaller in body size yet alive (Fig 1H). At E16.5, the DKO embryos harvested were lethal, infiltrated with blood and partially absorbed by the uterus (Fig 1I). Together, these data suggest that Ccny and Ccnyl1 have overlapping functions in development. As neither single mutant displays discernable mammary gland phenotype, functional redundancy likely persists during mammary development.

### *Ccnyl1* expression coincides with robust Wnt signaling activation in pubertal mammary glands

**Fig 2:**
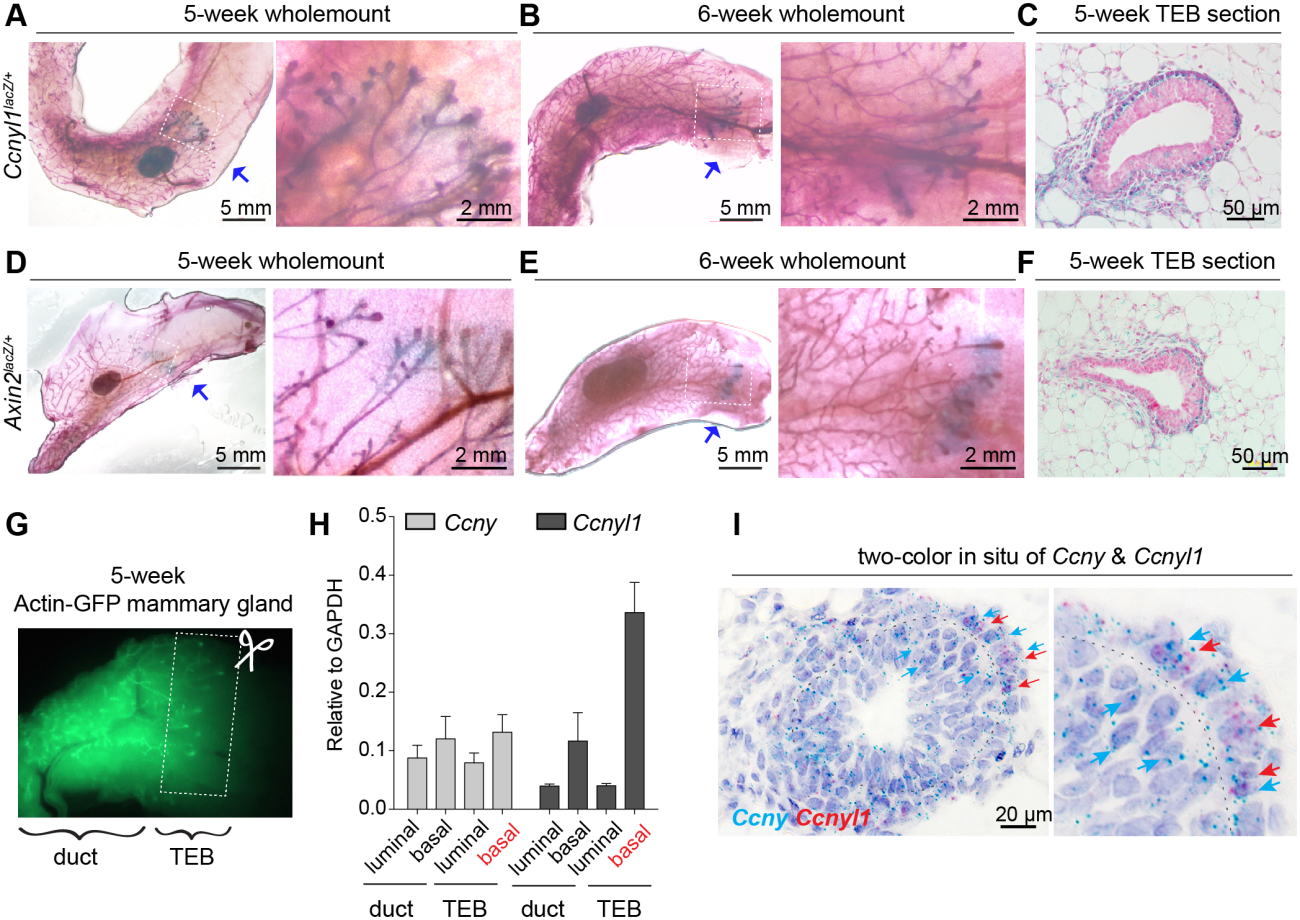
*Ccnyl1* expression coincides with robust Wnt signaling activation in the developing mammary gland. **(A-B)** Whole mount X-gal staining (blue) of mammary gland isolated from 5- week-old **(A)** or 6-week-old **(B)** *Ccnyl1*^*lacZ/+*^ female mice. The mammary glands were counterstained with carmine (red). Arrows indicate the robust X-gal staining in TEBs. Shown data are representative of three independent experiments. **(C)** Paraffin section of TEB from 5-week-old *Ccnyl1*^*lacZ/+*^ mouse. The nucleus was stained with nuclear fast red. X-gal staining (blue) is enriched in the basal epithelial cells and surrounding fibroblasts. **(D-E)** Whole mount X-gal staining (blue) of mammary gland isolated from 5- week-old **(D)** or 6-week-old **(E)** *Axin2*^*lacZ/+*^ female mice. The mammary glands were counterstained with carmine (red). Arrows indicate the robust X-gal staining in TEBs. Shown data are representative of three independent experiments. **(F)** Paraffin section of TEB from 5-week-old *Axin2*^*lacZ/+*^ mouse. The nucleus was stained with nuclear fast red. X-gal staining (in blue) is enriched in the basal epithelial cells and surrounding fibroblasts. **(G)** Illustration showing dissection of ducts and TEBs from a 5-week-old *Actin*-GFP mammary gland. **(H)** qPCR analysis of *Ccny* and *Ccnyl1* expression in FACS-isolated basal and luminal cells in TEBs and ducts. Data are pooled from two independent experiments. **(I)** Double *in situ* hybridization of *Ccny* mRNA (cyan) and *Ccnyl1* mRNA (red) in TEBs. *Ccnyl1* expressing cells (red arrows) are preferentially located in the basal layer, while *Ccny* expressing cells (cyan arrows) are more evenly distributed in basal and luminal cells.

We examined the expression of *Ccnyl1* in the mammary gland using *Ccnyl1*^*lacZ/+*^ mouse. Mammary glands were isolated from pubertal mice (5-week and 6-week old) for whole mount X-gal staining. At this stage, mammary epithelium undergoes active extension. Interestingly, *Ccnyl1* expression was enriched at the forefront of the pubertal mammary epithelium extension where TEBs are located (arrows in Fig 2A and 2B). *Ccnyl1* expression appeared mostly in basal cells and surrounding stromal cells, but rarely in the inner layer body cells (Fig 2C). It has been reported that several members of the Wnt family are expressed in the mammary gland at this stage [19–21], which could contribute to the proliferative state of TEBs. We examined the Wnt-responsiveness in pubertal mammary glands using *Axin2*^*lacZ/+*^ reporter mouse [22]. We found that *Axin2*-expressing cells are also enriched in the TEB area (Fig 2D and 2E), with robust staining in the basal cells and surrounding stromal cells (Fig 2F), exhibiting a similar pattern to the Ccnyl1-expressing cells. To address whether *Ccnyl1* is expressed in *Axin2*^+^ cells, double in situ hybridization were performed, revealing that *Axin2* and *Ccnyl1* frequently co-localized in basal cells of the TEBs (S3A Fig).

We next investigated whether *Ccny* expression also has a TEB enriched pattern. We harvested mammary glands from 5-week-old Actin-GFP mice, in which the forefront of the epithelium has extended slightly past the lymph node. Guided by the green fluorescence of GFP, we separated the TEB region from the ducts (illustrated in Fig 2G). Basal (Lin-, CD24+, CD29hi) and luminal (Lin-, CD24+, CD29lo) cells were isolated by FACS from the two compartments for quantitative PCR (qPCR) analysis. We found that *Ccny* was evenly expressed in the ducts and TEBs, with little difference between luminal and basal cells (Fig 2H). By contrast, *Ccnyl1* exhibited a higher expression in TEBs, especially in the basal cell of TEBs (Fig 2H), consistent with the observation in the *Ccnyl1*^*lacZ/+*^ reporter mice (see Fig 2A–2C). Double colored RNA *in situ* hybridization was then performed to validate *Ccny* and *Ccnyl1* expression in TEBs. We found that, consistent with the qPCR results, *Ccny* mRNA was detected in both basal and luminal cells, whereas *Ccnyl1* mRNA was predominantly distributed in basal cells (Fig 2I). In 8-week-old nulliparous mice, the mammary gland has ceased rapid proliferation and the TEB structure has vanished. At this stage, we detected very rare *Ccnyl1* expression in mature mammary ducts (S3B Fig), similar to the *Axin2-lacZ* expression pattern at this stage (S3B Fig) [12]. Thus, *Ccnyl1* is robustly expressed in the basal cell of TEBs, coinciding with Wnt/β-catenin signaling activation.

### *Ccnyl1* expression in mammary cells is cell cycle regulated

**Fig 3:**
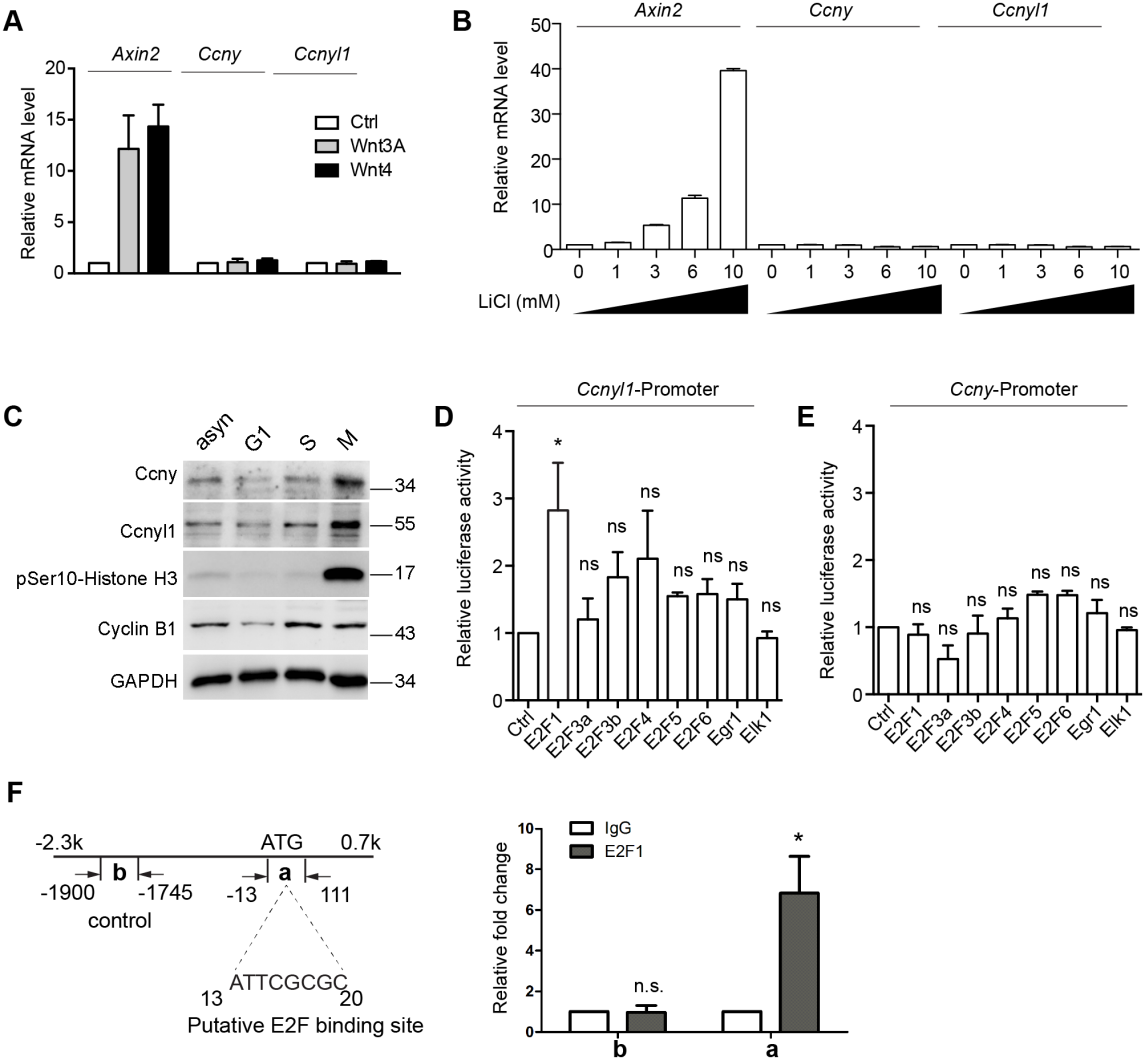
Ccnys expression is regulated by cell cycle but not Wnt signaling. **(A)** Wnt3A and Wnt4 failed to activate *Ccnys* expression in mammary basal cells. Primary basal cells (Lin^-^, CD24^+^, CD29^hi^) were isolated from 6-week old wildtype mice and cultured in Matrigel with Wnt3A protein, Wnt4 conditional medium or control medium for 6 days. qPCR analyses were performed for *Axin2*, *Ccny*, and *Ccnyl1*. Data are presented as mean±s.d from three independent experiments. **(B)** LiCl did not stimulate *Ccnys* expression in mammary basal cells. Basal cells were isolated from 6-week old wildtype mice, cultured in Matrigel and treated with LiCl at day 3. The cells were collected at day 6 for qPCR analyses. Representative results from three independent experiments are shown. Error bars are from technical repeats. Note that *Axin2* expression is activated by LiCl in a dose-dependent manner. **(C)** Expression of Ccnys during the cell cycle. Mouse mammary epithelial EpH4 cells were treated with mimosine to enrich G1 phase cells, thymidine to enrich S phase cells, or nocodazole followed by a shake-off to enrich mitotic cells. pSer10-Histone H3 was used to indicate M phase. Cyclin B1 was used to indicate the S phase and M phase. GAPDH served as loading control. **(D-E)** Luciferase assays for the *Ccnyl1* **(D)** and *Ccny* **(E)** promoters. The promoters were cloned into pGL4.17 vector to generate luciferase reporters. EpH4 cells were co-transfected with the reporter construct, pRL-TK, and an expression plasmid for each of the indicated transcription factors. After 48 h, the cells were lysed and examined for luciferase activity. Data are presented as mean±s.d from three independent experiments. Student’s *t* test: *p<0.05; ns, not significant. **(F)** EpH4 cells were subjected to chromatin immunoprecipitation (ChIP) using anti-E2F1 antibody or control rabbit IgG. qPCR was performed to detect the indicated regions in *Ccnyl1* promoter. Region **a** contains a putative E2F binding site. Region **b** contains an irrelevant sequence to serve as negative control. Data are presented as mean±s.d from three independent experiments. Student’s *t* test: *p<0.05.

In light of the overlapping expression of *Ccnyl1* and *Axin2* in pubertal mammary gland, we set to address whether the expression of *Ccnyl1* is induced by Wnt/β-catenin signaling. We cultured the basal cells in 3D matrigel as previously described [12] and found that neither Wnt3A nor Wnt4 (the endogenous Wnt in the mammary gland) was sufficient to induce *Ccnyl1* or *Ccny* expression, while either treatment successfully increased *Axin2* mRNA levels (Fig 3A). A gradient of lithium chloride (LiCl) was also used to activate Wnt signaling, yet it failed to stimulate *Ccnyl1* or *Ccny* expression (Fig 3B). Thus, Ccnys are likely not Wnt signaling targets.

Previous study indicates that Ccny is enriched in G2/M phase [9]. We thus examined whether Ccnyl1 level is regulated in a cell cycle dependent manner. We monitored endogenous Ccnyl1 level in drug synchronized mammary epithelial Eph4 cells and observed a prominent elevation of Ccnyl1 in M phase (Fig 3C). For insights into the upstream regulation of *Ccnyl1*, we examined possible binding sites for transcriptional factors in the *Ccnyl1* promoter region. The cell cycle related transcription factors E2Fs and proliferation related transcriptional factors, Egr1 and Elk1, were predicted to associate with the *Ccnyl1* promoter. We tested the potency of various transcription factors in activating *Ccnyl1* transcription by luciferase reporter assays. The -2.3kb to +0.7kb genomic region of *Ccnyl1* was cloned to drive a luciferase reporter gene. We found that E2F1 is able to activate the *Ccnyl1* promoter in Eph4 cells (Fig 3D). When similar luciferase assays were performed using the *Ccny* promoter, however, E2Fs could not activate Ccny-luciferase activities (Fig 3E). Furthermore, chromatin immunoprecipitation (ChIP) analysis confirmed direct association of E2F1 with the *Ccnyl1* genomic region containing a putative E2F1 binding site (Fig 3F). Together, these results suggest that *Ccnyl1* expression in mammary cells is cell cycle regulated.

### Ccnys are important for mammary stem/progenitor cell function *in vitro* and in mammary regeneration

**Fig 4:**
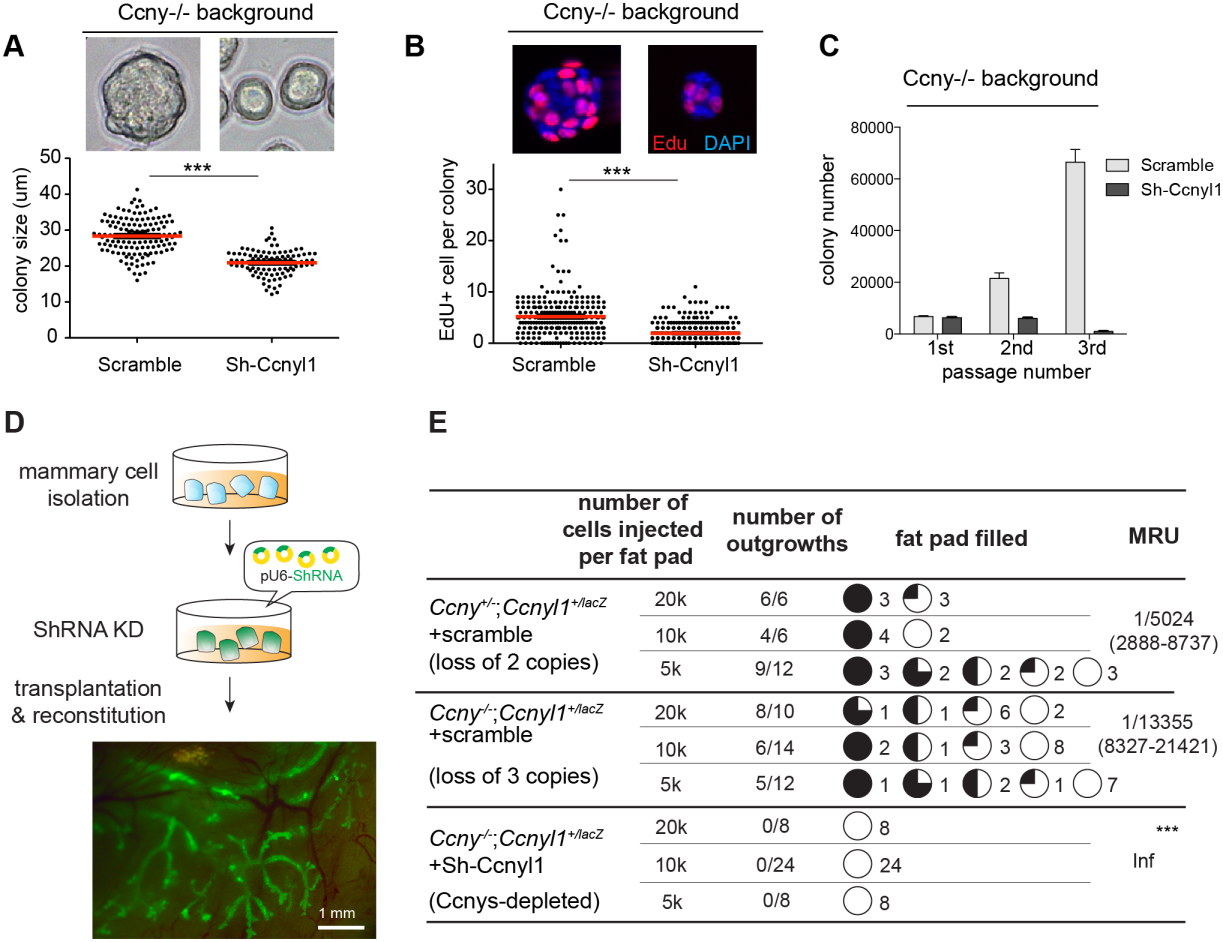
Depletion of Ccnys impedes basal stem/progenitor cell function *in vitro* and in regeneration. **(A)** *Ccny*^*-/-*^ mammary basal cells (Lin^-^,CD24^+^,CD29^hi^) were FACS-isolated from two 8-week-old mice, infected with lentiviral Scramble- or Ccnyl1-shRNA, and cultured in Matrigel. Colony sizes were measured at culture day 6. Shown data are representative of three independent experiments. Data are presented as mean±s.e.m. Student’s *t* test: ***p<0.001. **(B)** EdU labeling of basal colonies. 10 uM EdU of final concentration were added to the culture medium for 30 minutes. The colonies were then used for EdU staining. Data are presented as mean±s.e.m. Student’s *t* test: ***p<0.001. **(C)** Basal colonies were serially passaged and their numbers were quantified. **(D)** Illustration of the transplantation assays. A typical result of mammary outgrowth is shown. **(E)** All mammary epithelial cells were sorted out from 8 to 12-week-old mice and infected with lentivirus. After a 6-day culture, virally infected mammary cells of the indicated genotypes were FACS isolated based on GFP and transplanted by limiting dilution. Recipient fat pads were harvested at 8 weeks post transplantation. The mammary outgrowth numbers and sizes (shown as percentage of fat pad filled) are combined from three independent experiments. The mammary reconstitution unit (MRU) frequency is shown for each group. Inf represents infinitely small. Student’s *t*-test: *** p<0.001.

Axin2^+^ cells are enriched for basal mammary stem/progenitor cells [2]. In light of the similar expression patterns of Ccnyl1^+^ and Axin2^+^ cells, we next investigated the function of *Ccnys* on mammary stem/progenitor cells. We utilized the *Ccny* mutant mice (*Ccny*^-/-^) and knocked down the expression of *Ccnyl1* by shRNA in the *Ccny*^-/-^ background. The knockdown efficiency of the shRNA (Sh-Ccnyl1) was validated by Western analysis (S4 Fig). Basal cells were FACS-isolated from *Ccny*^-/-^ mammary glands and infected with control or Sh-Ccnyl1 lentivirus in suspension. Infected mammary cells were then cultured in 3D Matrigel to allow colony formation. We found that the colony sizes were significantly smaller when the Ccnys expression was inhibited (Fig 4A), with decreased cell proliferation as shown by reduced EdU incorporation (Fig 4B). Next we dissociated the primary colonies to single cells and replated them to examine serial colony formation. We found that knockdown of Ccnyl1 in *Ccny*^-/-^ mutant cells results in drastically decreased colony numbers in each passage, while the control colony numbers continuously expanded (Fig 4C). We also examined whether Ccnys affect luminal cell colony formation using the same approach in the *Ccny*^-/-^ background. No differences in luminal colony sizes were observed when Ccnyl1 was knocked down (S5 Fig). Together, these results show that *Ccnys* are important for the expansions of basal, but not luminal, colonies *in vitro*.

We next investigated the influence of *Ccnys* on the regenerative capacity of mammary stem/progenitor cells. We followed the in vivo knockdown method established in previous studies [23,24]. Mammary cells were isolated and infected with lentivirus to express both shRNA and GFP. Infected cells were FACS-isolated using the GFP tag, followed by transplantation into the cleared fat pad of immunocompromised recipient mice. The mammary outgrowths with GFP were examined at 8 weeks post transplantation (Fig 4D). In control experiments, we observed that *Ccny*^+/-^;*Ccnyl1*^lacZ/+^ mammary cells (loss of 2 copies of *Ccnys*) infected with a scramble shRNA readily generated outgrowths (Fig 4E). *Ccny*^-/-^;*Ccnyl1*^lacZ/+^ mammary cells (loss of 3 copies of *Ccnys*) infected with the scramble shRNA also generated new mammary glands though their outgrowths were smaller (Fig 4E). By contrast, *Ccny*^-/-^;*Ccnyl1*^*l*acZ/+^ cells infected with Sh-Ccnyl1 (loss of 3 copies of *Ccnys* plus RNAi) completely lost the regeneration capabilities and were not able to reconstitute any outgrowths (Fig 4E). Together, these results suggest that *Ccnys* are critical for mammary stem/progenitor cell self-renewal and regeneration capacity.

### Basal cells with *Ccnys* deficiency fail to contribute to normal development

**Fig 5:**
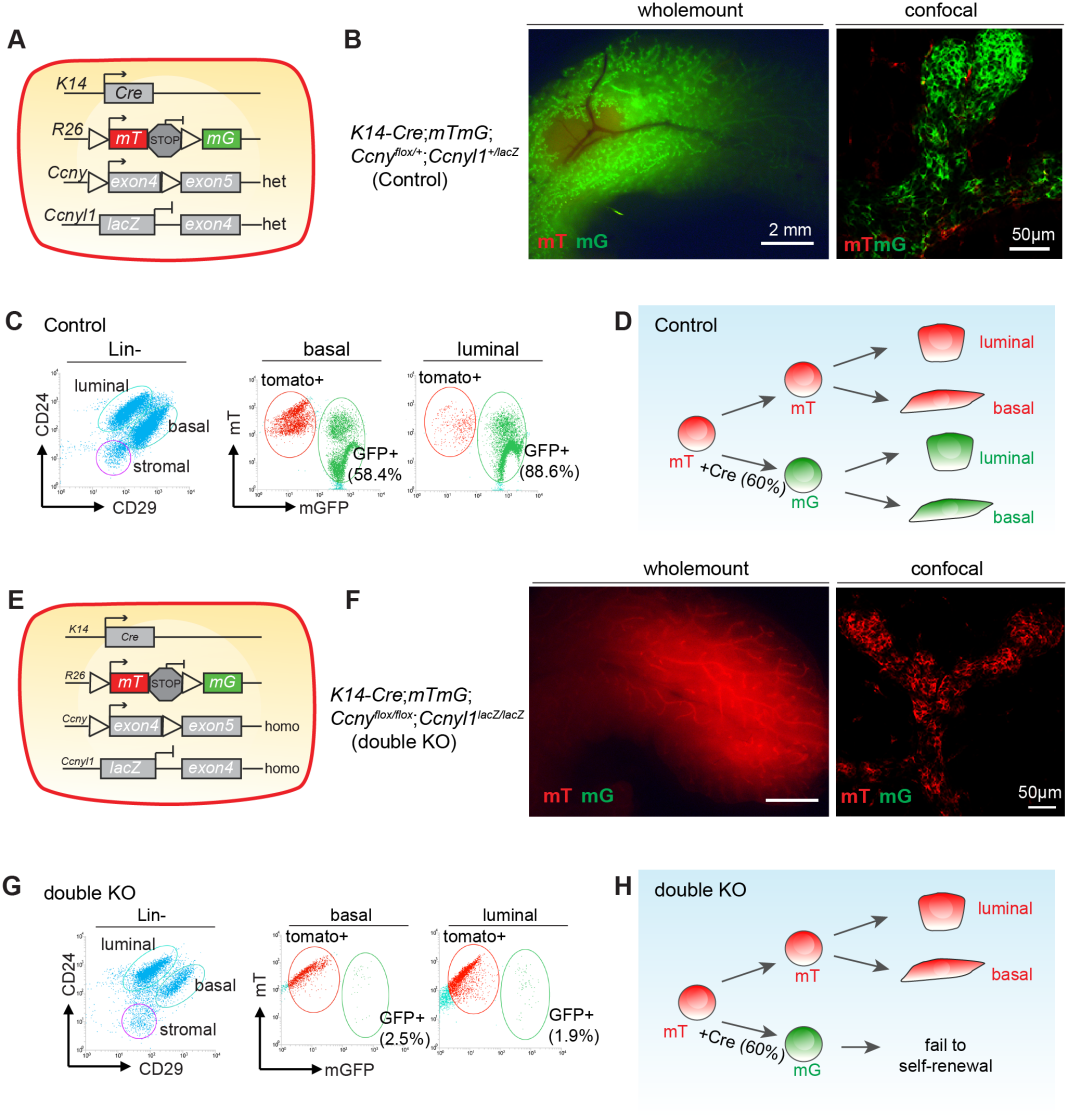
Basal cells with *Ccnys* deficiency fail to contribute to normal development. **(A)** Illustration of the control mouse genotypes. **(B)** Whole-mount analysis and confocal imaging of sections from the 8-week-old control mammary glands indicated that the majority of the cells were GFP-positive. n = 3 mice. **(C)** FACS analysis to quantify basal and luminal cell populations in a control mammary gland. 58.4% of basal cells (Lin^-^,CD24^+^,CD29^hi^) are GFP-positive, and 88.6% of luminal cells (Lin^-^,CD24^+^,CD29^low^) are GFP-positive. Shown data are representative of three independent experiments. **(D)** Illustration showing that wildtype stem cells with partial recombination result in chimeric mammary gland of GFP- and tdTomato-positive cells. **(E)** Illustration of the double knockout (DKO) mouse genotypes. **(F)** Whole-mount analysis and confocal imaging of sections from the 8-week-old DKO mammary glands indicated that most cells were tdTomato-positive. n = 3 mice. **(G)** FACS analysis to quantify basal and luminal cell populations in a DKO mammary gland. Only 2.5% of basal cells (Lin^-^,CD24^+^,CD29^hi^) are GFP positive, and 1.9% of luminal cells (Lin^-^,CD24^+^,CD29^low^) are GFP-positive. Shown data are representative of three independent experiments. **(H)** Illustration showing that Ccnys deficiency in stem/progenitor cells leads to self-renewal failure and loss in competition with wildtype stem cells.

To investigate the impact of *Ccnys* loss in normal development, we generated *K14-Cre*;*Ccny*^flox/flox^;*Ccnyl1*^lacZ/lacZ^;*mTmG* mouse model to delete both *Ccnys* using a basal cell specific *K14-Cre* [25], at the same time tracking the fate of the *Ccnys*-deficient cells using the mTmG reporter [26]. *K14* is activated as early as E15.5. The expressed Cre recombinase in basal cells would result in excision of the stop cassette in the reporter, thus marking the basal cells and their progeny, including luminal cells, with membrane-bound GFP (mG). On the other hand, intact cells would express membrane-bound tdTomato (mT), a red fluorescence protein. In control mice (*K14-Cre*;*Ccny*^flox/+^;*Ccnyl1*^lacZ/+^;*mTmG*) (Fig 5A), the mammary glands (8-week) were largely labeled with GFP, as evidenced by whole mount imaging as well as histological sections (Fig 5B). FACS analysis indicated that 58% of basal cells and 88% of luminal cells expressed GFP (Fig 5C and 5D), suggesting that the *K14-Cre* did not induce 100% recombination. By sharp contrast, the mammary glands of the *Ccnys*-deficient mice (*K14-Cre*;*Ccny*^flox/flox^;*Ccnyl1*^lacZ/lacZ^;*mTmG*) (Fig 5E) were mostly positive for tdTomato, as indicated by whole mount imaging and sections (Fig 5F). FACS analysis confirmed that GFP+ cells comprises of only 2.5% of basal cells and 2% of luminal cells (Fig 5G). In light of the partial efficacy of *K14*-Cre (58%) used in this study, we propose that stem cells that had successfully recombinated and lost all copies of *Ccnys* failed to compete with the normal stem cells in the developing mammary gland (Fig 5H). Since the mammary glands of *K14-Cre; Ccny*^flox/flox^;*Ccnyl1*^lacZ/lacZ^;*mTmG* mice exhibited normal morphology (S6 Fig), we postulated that the remaining normal stem cells are able to generate the whole mammary gland during the development and thus render the animals phenotypically silent. In fact, the replacement of cell population through cell competition can often be phenotypically silent as previous reported [27–29]. Together with the in vitro and transplantation data, these results suggest that loss of *Ccnys* impairs the function of mammary stem/progenitor cell, thereby affecting their contribution to differentiation during development.

### Forced activation of Wnt/β-catenin signaling rescues the Ccnys deficiency phenotype

**Fig 6:**
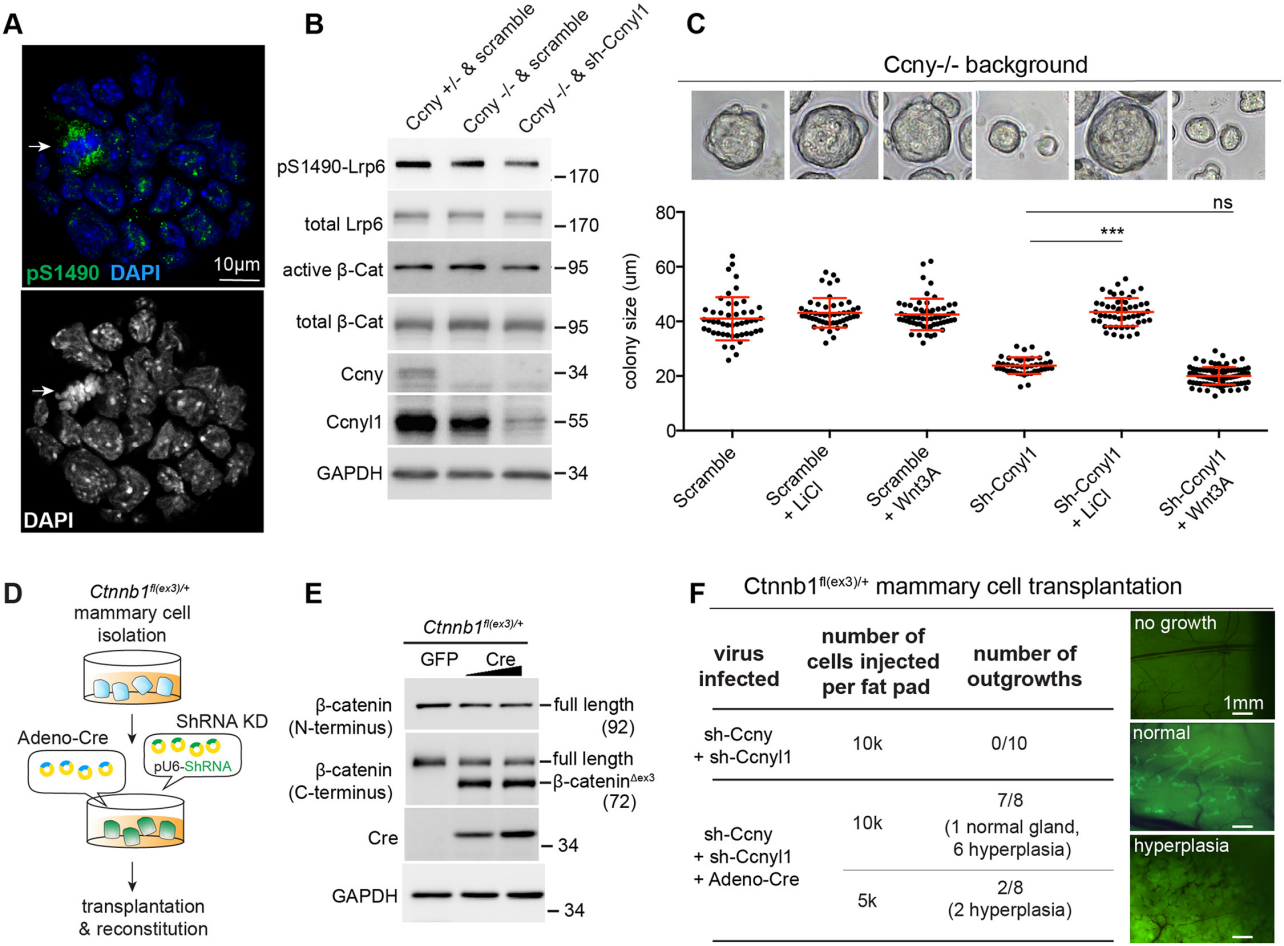
Constitutively active β-catenin rescues the Ccnys depletion-induced basal stem/progenitor cell defect. **(A)** Lrp6 Ser1490 phosphorylation staining in a basal colony. Basal cells were isolated from two 8-week old CD1 mice and culture in Matrigel for 6 days. Colonies were stained with anti-phospho-Ser1490 Lrp6 antibody (green). DNA was stained by DAPI (blue). The arrow indicates a mitotic cell in the colony. **(B)** Ccnys-depletion attenuates Lrp6 Ser1490 phosphorylation and β-Catenin activation in primary mammary cells. All mammary cells were isolated from 10-week-old *Ccny*^-/-^ mice and their *Ccny*^+/-^ littermate, respectively, and infected with scramble or sh-Ccnyl1 lentivirus. After 72 h of culture, the cells were treated with 200 ng ml^-1^ Wnt3A for another 16 h. Total cell lysates were then prepared and analyzed by western blotting. GAPDH served as loading control. Shown data are representative of three independent experiments.**(C)** LiCl restored the size of basal colonies to the normal range. Primary basal cells (Lin^-^,CD24^+^,CD29^hi^) were sorted from 8-week-old *Ccny*^-/-^ mammary glands, followed by infection of scramble or sh-Ccnyl1 lentivirus and culture in Matrigel. 200 ng ml^-1^ Wnt3A was added at day 1 or 3 mM LiCl was added at day 3. The colony sizes were measured at day 6. Data are presented as mean±s.e.m. Student’s *t* test: ***p<0.001. **(D)** Illustration of rescuing the regenerative capacity of Ccnys-deficient stem/progenitor cells by constitutively active β-catenin. All mammary epithelial cells were sorted out from 12-week-old *Ctnnb1*^*flox(ex3)/+*^ mice and virally infected with Adeno-Cre, Ccny-shRNA-mCherry and Ccnyl1-shRNA-GFP. GFP and mCherry double positive cells were FACS-isolated and transplanted. **(E)** Western analysis validating the generation of β-Catenin^Δex3^, a constitutively active form of β-Catenin, after Cre-mediated recombination. **(F)** *Ctnnb1*^*flox(ex3)/+*^ mammary cells were virally infected as indicated, followed by FACS-isolation and transplantation. Recipient fat pads were harvested at 8 weeks post transplantation and analyzed for mammary outgrowth. *Ctnnb1*^*flox(ex3)/+*^ cells with Ccnys-knockdown (infected with only the shRNAs) were not able to reconstitute any outgrowth, whereas *Ctnnb1*^*flox(ex3)/+*^ cells infected with both the shRNAs and Adeno-Cre efficiently generated outgrowth, often with hyperplasia formation. Representative images are shown. Results are combined from three independent experiments.

We next investigated the molecular mechanism by which Ccnys control the activities of mammary stem/progenitor cells. Ccny has been implicated in Lrp6 phosphorylation at Ser1490 (pS1490) in HEK293T cells [8,30]. In basal colonies, we observed enriched pS1490 in M phase cells (Fig 6A), suggesting that the cell cycle induced Lrp6 phosphorylation occurs during basal stem/progenitor cell expansion. To address the contribution of Ccnys in this event, we knocked down Ccnyl1 by shRNA in *Ccny*^*-/-*^ mammary cells in order to completely inhibit the expression of Ccnys. We observed that the levels of Lrp6 phosphorylation at S1490 and active form of β-catenin were reduced in these cells (Fig 6B). In embryonic fibroblasts (MEFs) isolated from the *Ccnys* DKO mice, similar reduction in pS1490 was also observed (S7 Fig). Thus Ccnys are important for the S1490 phosphorylation of Lrp6 and may function in mammary basal stem/progenitor cells through Lrp6 activation.

We tested whether activation of Wnt/β-catenin signaling can restore the phenotypes induced by the loss of Ccnys. As described in Fig 4, knockdown of Ccnyl1 in a *Ccny*^*-/-*^ background resulted in decreased basal colony sizes in 3D culture. We found that inhibiting GSK3β by LiCl restore the size of basal colonies to a normal range, while Wnt3A is ineffective (Fig 6C), supporting the notion that Ccnys function at the level of Lrp6 phosphorylation [9,11].

Next, we assessed the rescue capability in regeneration assays. We induced constitutive Wnt/β-catenin signaling activation using the *Ctnnb1*^*flox(ex3)/+*^ allele [31]. Mammary cells from *Ctnnb1*^*flox(ex3)/+*^ mice were infected with Cre adenovirus to induce recombination (Fig 6D). Simultaneously, we used mCherry-tagged Sh-Ccny and GFP-tagged Sh-Ccnyl1 lentivirus to knockdown expression of both Ccnys (Fig 6D; S4 Fig). The appearance of the N-terminal truncated form of stabilized β-catenin (β-catenin^Δex3^) (Fig 6E) indicated that Wnt signaling is constitutively activated in the Cre-expressed mammary cells. By contrast, no β-catenin^Δex3^ band was detected in the cells infected with a control adenovirus (Fig 6E). In order to assess their capability in regeneration, the infected cells were FACS-isolated and transplanted into the recipient cleared fat pads. We found that *Ctnnb1*^*flox(ex3)/+*^ mammary cells infected with the shRNAs were not able to reconstitute any outgrowth (Fig 6F), due to loss of Ccnys. When *Ctnnb1*^*flox(ex3)/+*^ mammary cells were infected with both the shRNAs and Adeno-Cre, however, they efficiently generated outgrowths, often with hyperplasia formation (Fig 6F), a phenotype reminiscent of activation of *Ctnnb1*^*flox(ex3)/+*^ alone [32], indicating that the constitutively active β-catenin restores the regenerative capability in the Ccnys-deficient stem/progenitor cells. Together, these data suggest that Ccnys’ regulation of Lrp6 activation facilitates Wnt/β-catenin signaling in mammary stem/progenitor cells.

## Discussion

### Redundancy of Ccny and Ccnyl1 in mouse development

Our study demonstrates the physiological significance of Ccnys in embryonic development and developing mammary gland. While the detailed cause of the embryonic lethality requires further investigation in the future, our results establish that mitosis-induced Wnt signaling enhancement is essential for keeping the properties of dividing mammary stem/progenitor cells. Although Ccny and Ccnyl1 showed different expression patterns in developing mammary gland (Fig 2), they are functionally redundant. In addition to single knockout (S2 Fig), female mice with only one allele of either *Ccny* or *Ccnyl1* were not only viable (Fig 1G) but also produced functional mammary glands. Only upon full knockout did the mammary cell fail to contribute to the mammary gland formation (Fig 5). Consistently, *Ccny*^-/-^;*Ccnyl1*^+/lacZ^ basal cells still reconstituted mammary outgrowths upon transplantation until the residual Ccnyl1 was further depleted by RNAi (Fig 4D and 4E). These results indicate that in mammals not only the gene numbers (two genes) but also the copy numbers (four alleles) are redundant for *Ccnys*, at least in most events of the development. This possibly serves as a way to safeguard this important layer of Wnt regulation.

### Cdk and cyclin as links between proliferation and stemness in cycling stem cells

The interconnections between cell cycle progression and cell fate specification have been explored in embryonic stem cells (ESCs) owing to the robust in vitro culture systems. The pluripotent status and differentiation propensity of ESCs are determined by specific cell-cycle profiles [1,33–35]. The importance of the cell cycle towards adult stem cells has also been documented in a variety of organs [2,36,37]. Notably, CDKs, together with their regulatory subunits cyclins, are involved in coupling cell cycle with stemness. G1 Cyclins, such as cyclin D, can impact the tendency and capacity of neural stem cells and hematopoietic stem cells to differentiate [37,38], and CDK6 regulates hematopoietic stem cell quiescence exit [39]. Our study adds a new episode by showing that the Ccnys-enhanced Wnt signaling activities in M phase is essential for dividing mammary stem/progenitor cell to maintain their competitiveness and developmental potential.

In adult stem cells, Wnt signaling activation can impact the adult stem cells by promoting cell-cycle entry through induced expression of the G1 factor, c-Myc and cyclin D1, which function as switches between quiescence and division (in intestine) [40,41]. Our data indicate that the reverse is also true, particularly the cell cycle can impact the adult stem cells by enhancing Wnt signaling activities during mitosis, which is crucial in maintaining the stem/progenitor cell properties and the progeny cell fate decision (in mammary gland). Our data highlight a cell cycle and Wnt signaling feed forward mechanism in active stem/progenitor cells for their expansion.

In many in vitro expansion systems of adult stem cell, both Wnts and mitogenic growth factors are required for sustaining the culture [12,42–45]. Wnt proteins are not required for proliferation in these contexts, indicating that inhibition of differentiation is its main function in self-renewal [12,46]. This leads to a simplified view that mitogenic growth factors are responsible for pushing the cells into division. In light of the feed forward mechanism, mitogenic growth factors also partake in keeping the stem/progenitor cell properties by influencing the output of Wnt signaling during cell division.

### Steps up the competition with higher Wnt signaling activation

Many parameters can trigger cell competition, including differences in protein synthesis rates, growth factor receptivity and the expression level of Myc [27]. The replacement of cell population through cell competition is phenotypically silent, because the competitor cells conform to size-control mechanisms [27–29]. In this study, we found that deletion of Ccnys in a subset of mammary stem/progenitor cells diminishes their capability in generation of progeny cells and contribution to development, due to loss in competition with wild type stem cells. Hence, the Wnt signaling enhancement mediated by Ccnys is critical for dividing stem/progenitor cells to retain their competitiveness and full potency. This process may occur naturally, as it might provide a mechanism for elimination of suboptimal stem/progenitor cells during development.

In conclusion, our findings here establish the importance of Ccnys in keeping the stem/progenitor cell properties and contribute to a better understanding of cell cycle control of Wnt signaling activation in cycling mammary stem/progenitor cells.

## Materials and Methods

### Ethics statement

All procedures were carried out in accordance with the Chinese guidelines for the care and use of laboratory animals. Experimental procedures were approved by the Animal Care and Use Committee of Shanghai Institute of Biochemistry and Cell Biology, Chinese Academy of Sciences (SIBCB-NAF-15-002-S335-005).

### Experimental animals

Experimental procedures were approved by the Animal Care and Use Committee of Shanghai Institute of Biochemistry and Cell Biology, Chinese Academy of Sciences. All mice were maintained in the specific-pathogen-free animal facility. The Ccny flox mice were constructed in Shanghai Research Center For Model Organisms. The Ccny flox mice were maintained on a 129/B6 mixed background. The Ccnyl1 knockout-first (kof) mice were kindly provided by EMMA (Stain ID, EM:04396. Stain name, C57BL/6NTac-Ccnyl1<tm1a(EUCOMM)Wtsi>/H), and maintained on a C57BL/6 background. The *Axin2*^*lacZ/+*^ mice, *K14-Cre* mice, *Rosa26*^*mTmG*/+^mice and *Ctnnb1*^*fl(ex3)/+*^ mice were described previously [22,26,31,47]. The *EIIa-Cre* mice were maintained on a FVB background. Genotyping analyses were performed by PCR with genomic DNA extracted from tail tips. For embryos studies, pregnancies were obtained by natural mating and were timed from the day of the vaginal plug, which was defined as embryonic day (E) 0.5. Embryos were dissected from uterus and then photographed under a dissection microscopy (Olympus SZX16). Primary mouse embryonic fibroblasts (MEFs) were isolated from E14.5 embryos and cultured as previously described [48].

### Plasmids

The full lengths of mouse *Ccny* (NM_026484.3) and *Ccnyl1* (ENSMUSG00000070871) cDNAs were amplified by PCR. Ccny and Ccnyl1 cDNA was cloned into pcDNA-HA to express HA-Ccny and HA-Ccnyl1 fusion proteins. To express GST or 6×His tagged Ccnyl1, its cDNA was cloned into pGEX-4T-1 or pET28a vector. For lentiviral shRNA constructs, the annealed oligonucleotides were inserted into pLKO.1, modified by replacing the puromycin-resistance gene with a cDNA encoding GFP or mCherry. The sequences of shRNAs were as follows: Scramble shRNA: 5'-TCCTAAGGTTAAGTCGCCCTCG-3'; *Ccnyl1* shRNA: 5'-GCTCATGCTCAACAATATTTC-3'; Ccny shRNA: 5'-GCAAGAGTCTCTTCATTAACCC-3'.

### Antibodies

GST tagged Ccnyl1 (77–367 aa) and 6×His tagged Ccnyl1 (77–367 aa) were expressed in *E*.*Coli* BL21 codon plus strain for anti-Ccnyl1 antibody generation and purification. Anti-Ccny antibody was as prepared previously described [10]. Other antibodies used were as follows: anti-β-catenin antibody (C-terminus) (BD, 610153); anti-β-catenin antibody (N-terminus) (Abclonal, A2064); anti-active β-catenin antibody (Millipore, 05–665); anti-Cre antibody (Novagen, 69050–3); anti-GAPDH antibody (Proteintech, 10494-1-AP); anti-Flag antibody (Sigma, F3165); anti-β-actin antibody (Sigma, 5316); anti-Lrp6 antibody (Cell Signaling Technology, 3395S); anti-phospho-Histone H3 Ser10 antibody (Cell Signaling Technology, 3377S); anti-phospho Lrp6 Ser1490 antibody for western blot (Cell Signaling Technology, 2568); anti-phospho Lrp6 Ser1490 antibody for immunofluorescence (gift from Christof Niehrs lab); anti-E2F1 IgG (Santa Cruz, sc-193).

### X-gal staining and whole mount analyses of mammary glands

Mammary glands were dissected and stained with X-gal as described [49]. Briefly, mammary glands were dissected and washed once with PBS. Mammary glands were fixed at room temperature for 2 h with β-galactosidase fixative (0.2% glutaraldehyde, 1.5% formaldehyde, 5 mM EGTA, 2 mM MgCl_2_ in PBS), and then washed 3 times in wash buffer (2 mM MgCl_2_, 0.01% sodium deoxycholate and 0.02% Nonidet P-40 in PBS) for 15 min each time. Finally, mammary glands were stained overnight in staining solution (1 mg/ml X-gal, 5 mM K_4_Fe(CN)_6_, 5 mM K_3_Fe(CN)_6_ and 2 mM MgCl_2_ in PBS) at 30°C. After X-gal staining, mammary glands were washed with PBS for several times, stained with carmine alum, and then dehydrated in 50%, 70%, 95%, 100%, 100% ethanol and Histoclear. Whole mount analyses were performed under a dissection microscope (Leica). To localize lacZ^+^ cells easily, mammary glands embedded in paraffin were sectioned for 10 μm thick. The sections were de-waxed in Histoclear, rehydrated in 100%, 95%, 85%, 75%, 50%, 30% ethanol, and counterstained with nuclear fast red, and followed by a serial of dehydration in 30%, 50%, 75%, 85%, 95%, 100% ethanol, and cleared in Histoclear before sealed by coverslip. The stained samples were photographed with an Olympus BX51 microscope equipped with an Olympus DP71 cooled CCD camera.

For whole mount analyses in conditional knockout mice (cKO), observation was made under a fluorescence dissection microscopy (Leica). After analysis, the mammary glands were processed for frozen sections or FACS analysis.

### In situ hybridization

The terminal end-buds of mammary glands from 5 week-old female mice were fixed with 10% neutral buffered formalin at room temperature for 36 h and then prepared for paraffin sections of 7 μm thick. In situ hybridization was performed using the RNAscope kit (Advanced Cell Diagnostics) following the manufacturer’s instructions. *Axin2*, *Ccny* and *Ccnyl1* probes were ordered from Advanced Cell Diagnostics.

### Primary mammary cell preparation, cell labeling and flow cytometry

Mammary glands were isolated from 8- to 12-week-old virgin or other specified-stage female mice. The minced tissue was placed in culture medium (RPMI 1640 with 25 mM HEPES, 5% fetal bovine serum, 1% penicillin-streptomycin-glutamine, 300 U ml^−1^ collagenase III (Worthington)) and digested for 2 h at 37°C. After lysis of the red blood cells in NH_4_Cl, a single-cell suspension was obtained by sequential incubation with 0.25% trypsin-EDTA at 37°C for 5 min and 0.1 mg ml^−1^ DNase I (Sigma) for 5 min with gentle pipetting, followed by filtration through 70-μm cell strainers. For cell labeling, the following antibodies were used: FITC-conjugated CD31, CD45, TER119 (BD PharMingen); CD24-PE/cy5, CD29-APC (Biolegend). Antibody incubation was performed on ice for 15 min in HBSS with 10% fetal bovine serum. All sortings were performed using a FACSJazz (Becton Dickinson). The purity of sorted population was routinely checked and ensured to be more than 95%.

### In vitro colony formation

FACS-sorted cells were infected with lentivirus overnight, and resuspended at a density of 4 × 10^5^ cells ml^−1^ in chilled 100% growth-factor-reduced Matrigel (BD Bioscience). The mixture was allowed to polymerize before being covered with culture medium [DMEM/F12, ITS (1:100; Sigma)] which was changed every 24 h. Primary colony numbers were counted and the diameters were measured after 5–7 days in culture. The colonies were mostly spherical, if colony was oval, the long axis was measured. LiCl (Sigma) was added to the culture medium from day 3. For Wnt treatment, 200 ng ml^−1^ Wnt3A or Wnt4 conditional medium (1:50 conditional medium from Wnt4-expressing EpH4 stable cell line) was added from day 1. For passaging colonies, the medium was aspirated, and Matrigel was digested by incubation in 500 μl of Matrigel recovery solution (BD Bioscience) for 1 h on ice. Colonies released from Matrigel were harvested after pelleting. Single cells were obtained through incubation in 0.25% Trypsin-EDTA for 5 min at 37°C followed by gentle pipetting. Single cells were then replated in Matrigel as described above.

### Mammary fat pad transplantation

Mammary cells were prepared, infected by indicated virus, and then cultured in monolayer. After 5–7 days, cells were digested with trypsin, sorted by FACS, and resuspended in 50% Matrigel, PBS with 20% FBS, and 0.04% Trypan Blue (Sigma), to be injected in 10-μl volumes into the pre-cleared fat pads of 3-week-old female nude mice. Reconstituted mammary glands were harvested after 8–10 weeks post surgery. Outgrowths were detected under a fluorescence dissection microscope (Leica). Outgrowths with more than 10% of the host fat pad filled were scored as positive.

### Cell culture, transfection and cell cycle synchronization

HEK293T cells, AD-293 cells, and EpH4 cells were cultured in DMEM (high glucose, Hyclone) supplemented with 10% fetal bovine serum, 100 units/ml streptomycin, 100 units/ml penicillin, and 0.3 mg/ml L-glutamine at 37°C and 5% CO_2_.

Plasmids were prepared using UNIQ-500 (Sangong Biotech). Lentivirus was packaged in HEK293T cells as described [50]. Briefly, HEK293T cells were co-transfected with vesicular stomatitis virus G, packaging plasmid Delta8.9, and transfer vector, using the conventional calcium phosphate method. At 48 h post-transfection, the culture medium was harvested and prepared for ultracentrifugation. The pellet of lentivirus was resuspended in PBS. Adenovirus particles were prepared in AD-293 cells and concentrated using CsCl gradient centrifugation as described [51]. Purified virus particles were stored at -80°C.

EpH4 cells were synchronized to specific cell cycle stage by drug treatment. Briefly, cells were synchronized to late G1 phase by 800 μM mimosine treatment for 18 h or to S phase by administration of 2 mM thymidine for 18 h. Mitotic cells were harvested by shake-off after 200 ng ml^−1^ nocodazole treatment for 4 hrs.

### Cell fractionation

Cell fractionation was performed using the Membrane and Cytosol Protein Extration Kit (Beyotime, P0033). Briefly, unsynchronized EpH4 cells were lysed, and nucleus was depleted by centrifugation for 10 min at 700 *g* at 4°C. The post-nuclear supernatant of EpH4 cells was fractionated by ultracentrifugation for 30 min at 14,000 ×*g* at 4°C into cytosol (C) and membranes (M). Equal-volume fractions of C and M were analyzed by western blotting.

### Quantitative RT-PCR

Total RNA was extracted using RNAiso plus (Takara), and the PrimeScript RT Master mix kit (Takara) with oligo(dT) primers was used for the reverse transcription reaction. Quantitative RT-PCR (qPCR) was performed using an Applied Biosystems 7500HT sequence detection system with a FastStart Universal SYBR Green Master Mix kit (Roche). *Gapdh* served as internal control. The reaction mixtures were incubated at 95°C for 10 min, followed by 40 cycles of 15 s at 95°C and 1 min at 60°C. qPCR primers used in this work:

*Ccny-F*: 5'-TCTCTTCATTAACCATCATCCTCC-3',

*Ccny-*R: 5'-AATTTGCTTCTGTTCTGGGT-3';

*Ccnyl1-*F: 5'-AGTGACGTTGGTTTACTTAGAG-3',

*Ccnyl1-R*: 5'-GCCTTTCCATCTCATTCATGTC-3';

*Axin2-F*: 5’-AGCCTAAAGGTCTTATGTGGCTA-3’

*Axin2-R*: 5’- ACCTACGTGATAAGGATTGACT-3’

*Gapdh-F*: 5'-AGGTCGGTGTGAACGGATTTG-3',

*Gapdh-R*: 5'-TGTAGACCATGTAGTTGAGGTCA-3'.

### Western blot

Tissues or cells were lysed with ice-cold RIPA buffer [50 mM Tris-HCl (pH7.5), 150 mM NaCl, 1% Nonidet P-40, 0.5% deoxysodium cholate, 0.1% SDS, 5 mM EDTA, 10 mM NaF. Before use, add 1 mM PMSF, 3 mM dithiothreitol, 1 mM sodium vanadate, and protease inhibitors (Merck)]. Proteins were resolved by SDS-PAGE and transferred to nitrocellulose membranes or polyvinylidene fluoride membranes. Immunoblots were developed in chemiluminescence reagent (PerkinElmer Life Sciences) and exposed in a Fujifilm LAS 4000 imager.

### Fluorescent microscopy

Antibodies were diluted in PBS containing 2% BSA. Primary colonies were fixed with 4% paraformaldehyde at room temperature for 10 min. Colonies were then blocked in PBS containing 0.2% Triton X-100 and 10% goat serum for 1 h, followed by incubation with rabbit anti-phospho-Lrp6 Ser1490 antibody overnight at 4°C. The samples were washed with PBS containing 2% BSA for 3 times and incubated with secondary antibody for 1 hr. The samples were finally washed with PBS for 3 times and stained with DAPI. Whole mount fluorescent images of mammary glands were obtained using a Leica MZFLIII dissection microscope. RNA in situ images were acquired using a Zeiss A1-AXIO upright microscope. For confocal imaging, mammary glands were minced and then coverslipped. Confocal Images were acquired through a 40× or 63× oil immersion objective on a Leica TCS SP8 confocal microscope.

### Luciferase assay

EpH4 cells were plated into 24 well tissue culture dishes. After 16–20 h, EpH4 cells were co-transfected with 0.33 μg *firefly* luciferase reporter plasmid (pGL4.17) containing the promoter region of Ccnyl1, 0.033 μg *Renilla* luciferase plasmid (pRL-TK) and 0.67 μg pcDNA3-HA-E2Fs or the other transcription factors in per well of the 24-well plate. After 48 h, the cells were lysed and luciferase activities were measured with Dual-Luciferase Reporter Assay System (Promega, Madison, USA).

### Chromatin immunoprecipitation (ChIP) and PCR

ChIP analysis was performed as previously described [52]. Briefly, EpH4 cells were cross-linked with 1% formaldehyde for 10 min at room temperature. For each group, a 10-cm dish of EpH4 cells with 80% confluency were lysed and chromatin was sonicated in a sonicator for 30 min (with 7-sec sonication and 7-sec rest alternatively). Sonicated chromatin was then diluted and immunoprecipitated with anti-E2F1 IgG or rabbit normal IgG (Santa Cruz, sc-2027). Immunoprecipitation products and input were analyzed by quantitative PCR using the following specific primers:

Site a,

forward:5’-CAGCTCGAGATGAATGGAAACC-3’,

reverse: 5’-TAGCCAATCAGACCCGGACTTC-3’.

Site b,

forward: 5’-CAGCAATGTCTCCATGTCACAT-3’,

reverse: 5’-CCCATGAGCACAACACAATTTC-3’.

### Statistics

Results are presented as mean ± s.d., unless otherwise stated. Differences were considered significant when *P*<0.05 in an unpaired Student’s *t*-test. Three independent experiments were carried out for statistic results unless specified otherwise.

## Supporting Information

S1 Fig Alignment of Ccny and Ccnyl1, and validation of the Ccny and Ccnyl1 antibody specificity. (**A**) Protein sequence alignment of mouse Ccny and Ccnyl1. (**B-D**) Polyclonal antibodies were raised using full-length mouse Ccny and the 77–367 aa fragment of mouse Ccnyl1. HEK293T cells were transfected with pcDNA3-HA-Luciferase, pcDNA3-HA-Ccny or pcDNA3-HA-Ccnyl1 for 48 h. Total cell lysates were prepared and used for western blotting with anti-HA (**B**), anti-Ccny (**C**), or anti-Ccnyl1 (**D**) antibody. Weak cross-reactions were observed. Note that the MWs of mouse Ccny and Ccnyl1 are approximately 36 and 55 KD, respectively.
(TIF)

S2 Fig Ccny and Ccnyl1 play overlapping roles in mammary gland development. Representative whole-mount carmine staining of mammary glands from 7-week-old wildtype, Ccny^-/-^ and Ccnyl1^lacZ/lacZ^ mice.
(TIF)

S3 Fig *Ccnyl1* and *Axin2* expressions coincide in the developing mammary gland. (**A**) Two-color in situ hybridization of *Ccnyl1* (red) and *Axin2* (Cyan) mRNAs in the terminal end bud (TEB) of 5-week-old mammary gland. The arrows indicate representative basal cells with both *Ccnyl1* and *Axin2* expression. (**B**) X-gal staining of paraffin sections of 8-week-old *Ccnyl1*^*lacZ/+*^ or *Axin2*^*lacZ/+*^ mammary glands. X-gal staining signals (blue, arrows) indicate the expression of *Ccnyl1* or *Axin2* in few basal cells. The nucleus was counterstained with nuclear fast red.
(TIF)

S4 Fig Validation of the knockdown efficiency of Ccny1 and Ccny shRNAs. (**A**) HEK293T cells were co-transfected with pcDNA3-HA-Ccnyl1 and pLKO.1-GFP-Ccnyl1shRNA or pLKO.1-GFP-scamble shRNA. After 48 h, the cells were lysed and subjected to western blot analysis with anti-HA antibody. GAPDH served as loading control. (**B**) HEK293T cells were co-transfected with pcDNA-HA-Ccny and pLKO.1-mCherry-Ccny shRNA or pLKO.1-mCherry-scamble shRNA. After 48 h, the cells were subjected to western blot analysis with anti-HA antibody. GAPDH served as loading control.
(TIF)

S5 Fig Ccnys do not affect luminal colony growth. Luminal cells (Lin^-^,CD24^+^,CD29^low^) were isolated from 8-week-old *Ccny*^*-/-*^ mammary glands infected with Scramble or sh-Ccnyl1 lentivirus, and then cultured in Matrigel. Colony size was measured at day 6. Student’s *t*-test: n.s., not significant.
(TIF)

S6 Fig Ccny and Ccnyl1 cKO using K14-Cre is phenotypically silent. (**A-B**) whole-mount carmine staining of mammary glands from 11-week-old control (*K14-Cre;Ccny*^*flox/+*^;*Ccnyl1*^*lacZ/+*^;*mTmG*) **(A)** and cKO (*K14-Cre;Ccny*^*flox/flox*^;*Ccnyl1*^*lacZ/lacZ*^;*mTmG*) **(B)** mice.
(TIF)

S7 Fig Western analysis on Ccny and Ccnyl1 DKO MEFs. MEFs were isolated from Ccny heterozygous mutant and Ccnys DKO mouse embryos. Western analyses were performed to detect the indicated proteins. α-Tubulin served as loading control.
(TIF)
