# Supplementary figures and images for: Essential Roles of Cyclin Y-Like 1 and Cyclin Y in Dividing Wnt-Responsive Mammary Stem/Progenitor Cells
Source: PLoS Genet. 2016 May 20;12(5):e1006055. doi: 10.1371/journal.pgen.1006055 (PMC4874687; doi:10.1371/journal.pgen.1006055)

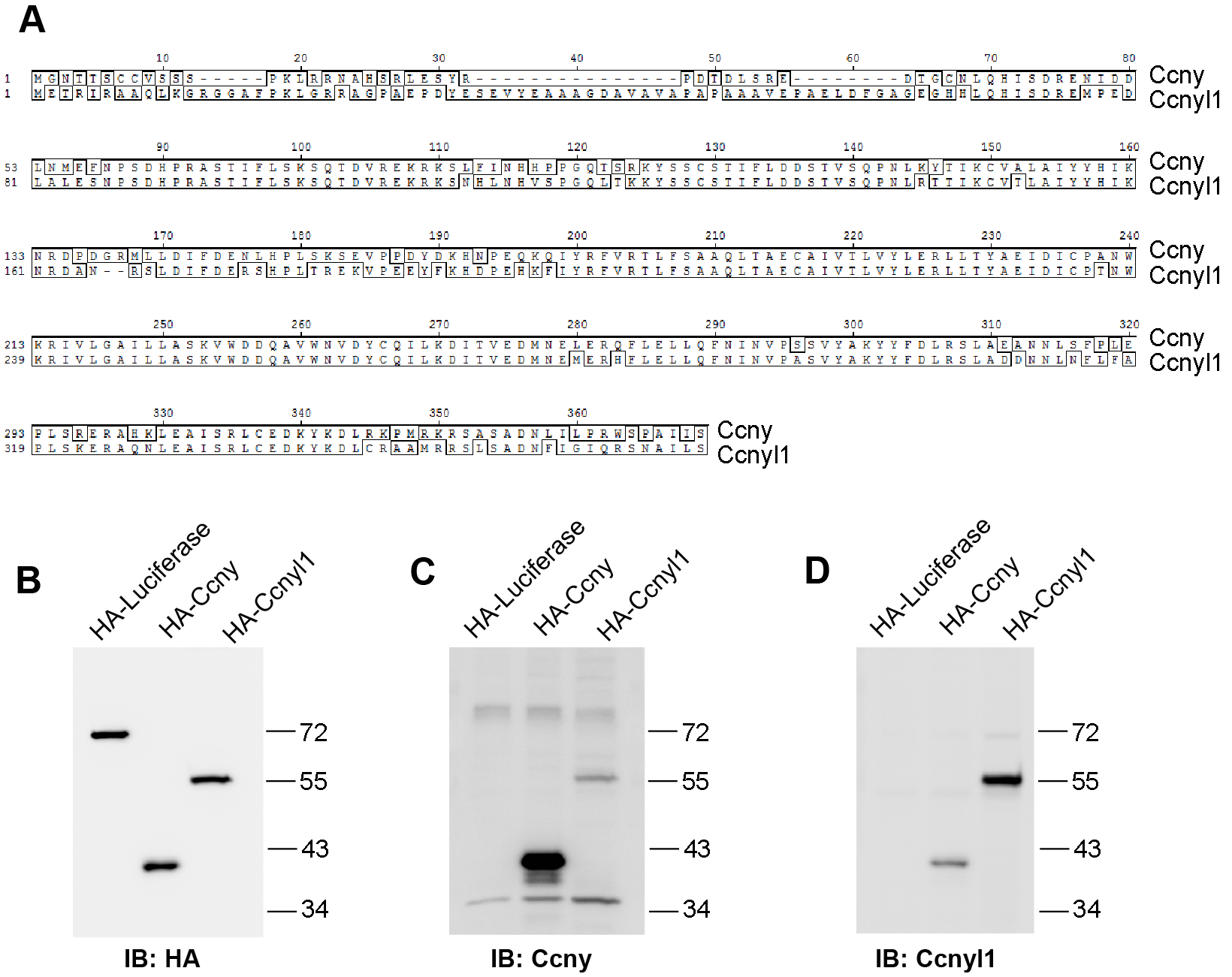

Supplement: S1 Fig — (A) Protein sequence alignment of mouse Ccny and Ccnyl1. (B-D) Polyclonal antibodies were raised using full-length mouse Ccny and the 77–367 aa fragment of mouse Ccnyl1. HEK293T cells were transfected with pcDNA3-HA-Luciferase, pcDNA3-HA-Ccny or pcDNA3-HA-Ccnyl1 for 48 h. Total cell lysates were prepared and used for western blotting with anti-HA (B), anti-Ccny (C), or anti-Ccnyl1 (D) antibody. Weak cross-reactions were observed. Note that the MWs of mouse Ccny and Ccnyl1 are approximately 36 and 55 KD, respectively. (TIF) [file pgen.1006055.s001.tif]

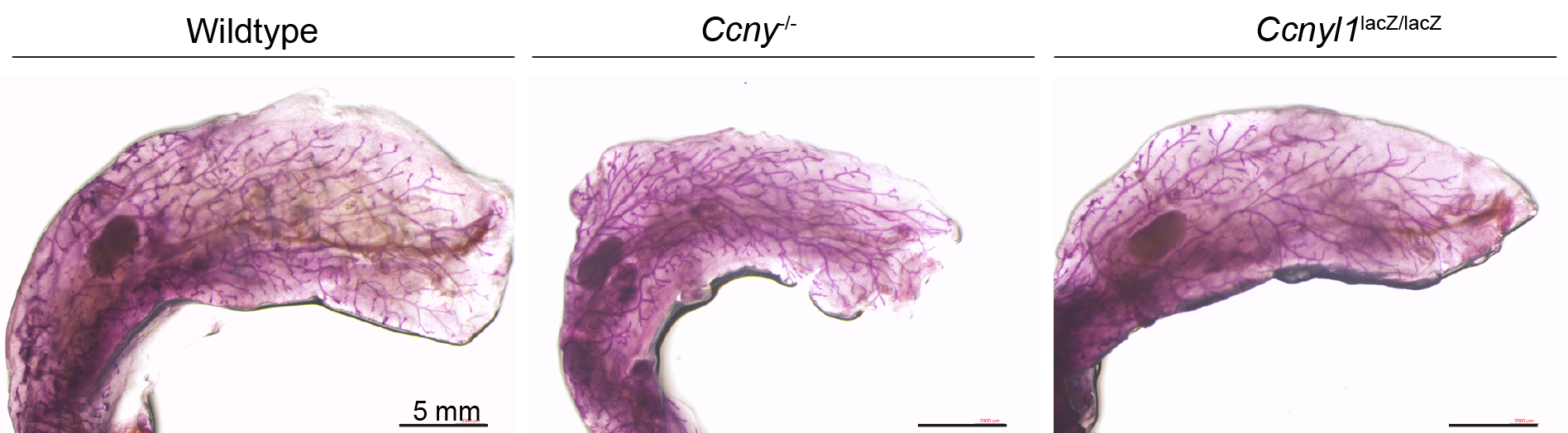

Supplement: S2 Fig — Representative whole-mount carmine staining of mammary glands from 7-week-old wildtype, Ccny-/- and Ccnyl1lacZ/lacZ mice. (TIF) [file pgen.1006055.s002.tif]

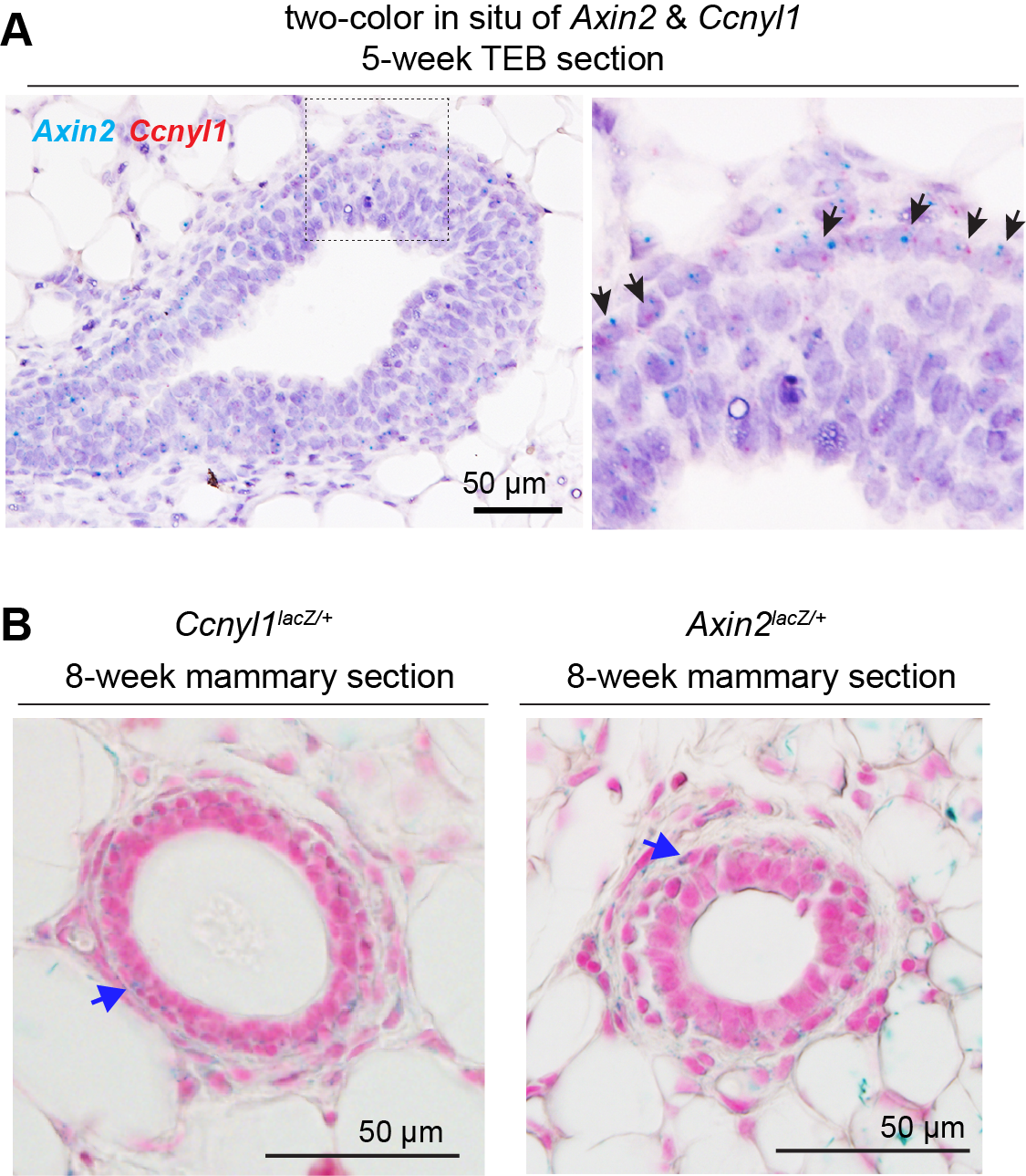

Supplement: S3 Fig — (A) Two-color in situ hybridization of Ccnyl1 (red) and Axin2 (Cyan) mRNAs in the terminal end bud (TEB) of 5-week-old mammary gland. The arrows indicate representative basal cells with both Ccnyl1 and Axin2 expression. (B) X-gal staining of paraffin sections of 8-week-old Ccnyl1lacZ/+ or Axin2lacZ/+ mammary glands. X-gal staining signals (blue, arrows) indicate the expression of Ccnyl1 or Axin2 in few basal cells. The nucleus was counterstained with nuclear fast red. (TIF) [file pgen.1006055.s003.tif]

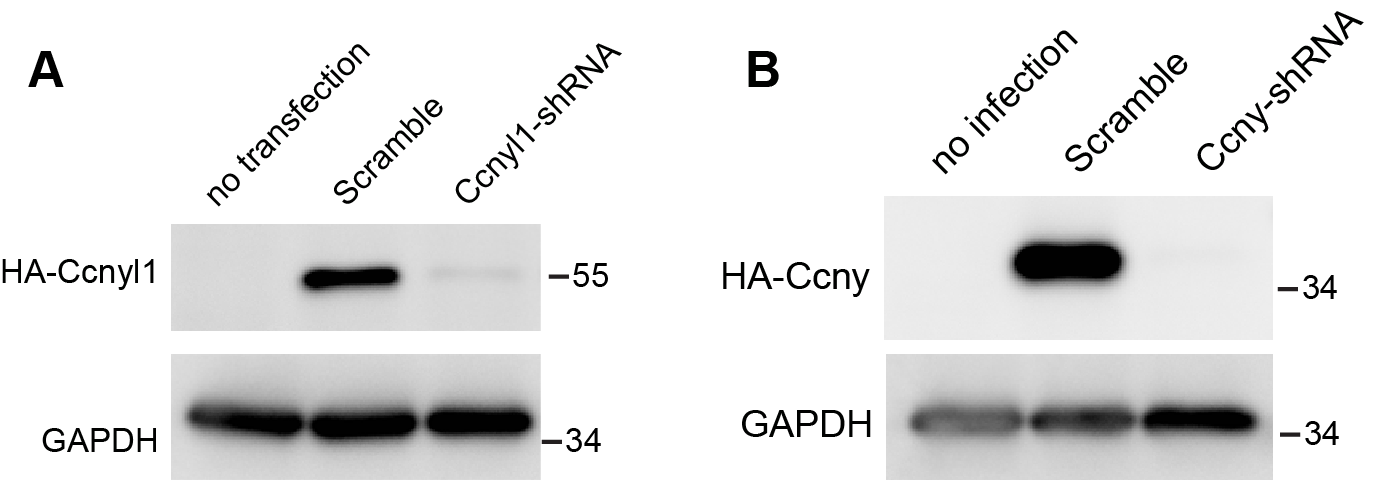

Supplement: S4 Fig — (A) HEK293T cells were co-transfected with pcDNA3-HA-Ccnyl1 and pLKO.1-GFP-Ccnyl1shRNA or pLKO.1-GFP-scamble shRNA. After 48 h, the cells were lysed and subjected to western blot analysis with anti-HA antibody. GAPDH served as loading control. (B) HEK293T cells were co-transfected with pcDNA-HA-Ccny and pLKO.1-mCherry-Ccny shRNA or pLKO.1-mCherry-scamble shRNA. After 48 h, the cells were subjected to western blot analysis with anti-HA antibody. GAPDH served as loading control. (TIF) [file pgen.1006055.s004.tif]

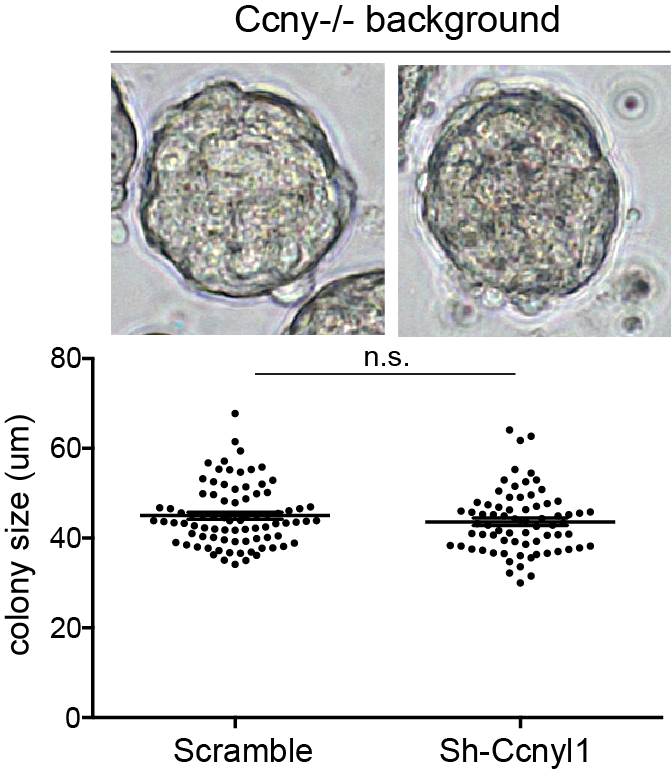

Supplement: S5 Fig — Luminal cells (Lin-,CD24+,CD29low) were isolated from 8-week-old Ccny-/- mammary glands infected with Scramble or sh-Ccnyl1 lentivirus, and then cultured in Matrigel. Colony size was measured at day 6. Student’s t-test: n.s., not significant. (TIF) [file pgen.1006055.s005.tif]

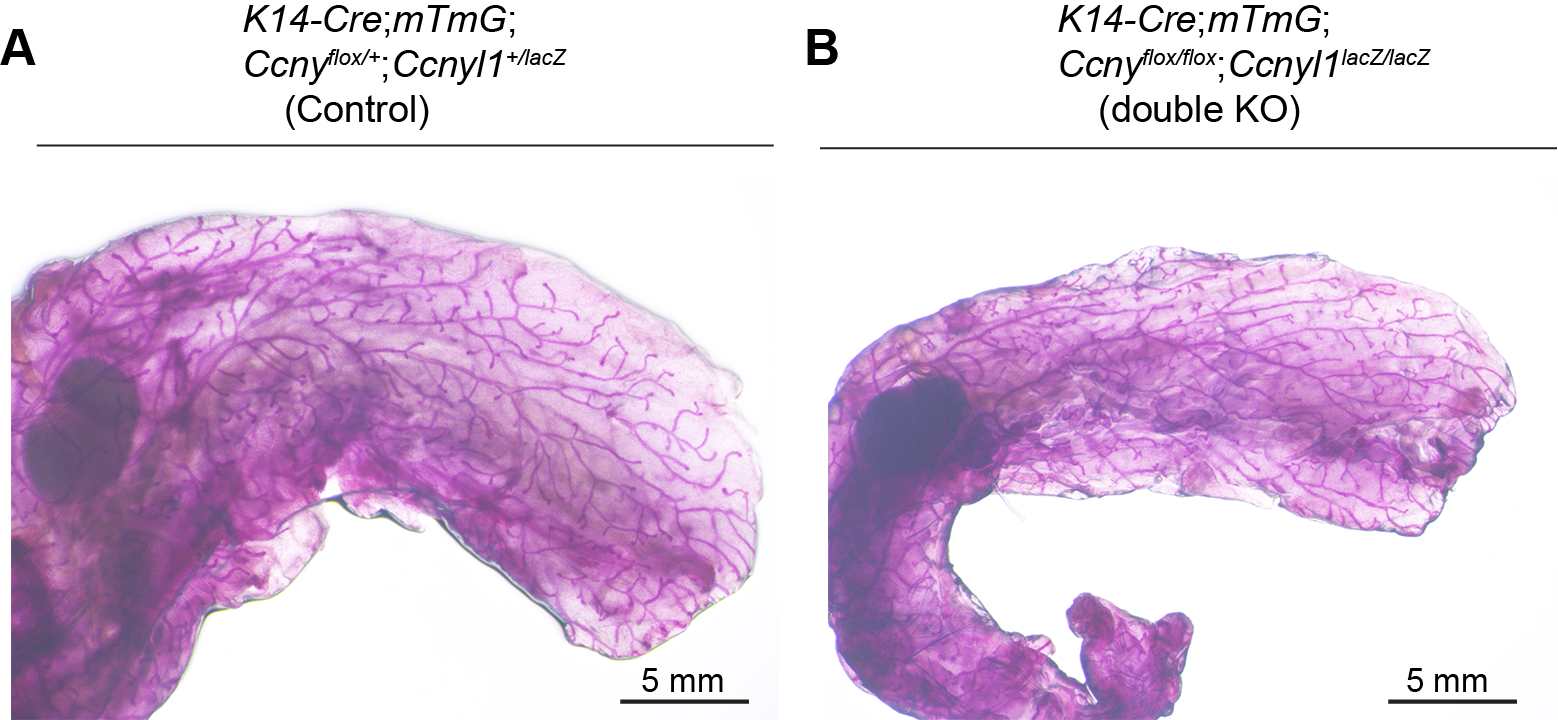

Supplement: S6 Fig — (A-B) whole-mount carmine staining of mammary glands from 11-week-old control (K14-Cre;Ccnyflox/+;Ccnyl1lacZ/+;mTmG) (A) and cKO (K14-Cre;Ccnyflox/flox;Ccnyl1lacZ/lacZ;mTmG) (B) mice. (TIF) [file pgen.1006055.s006.tif]

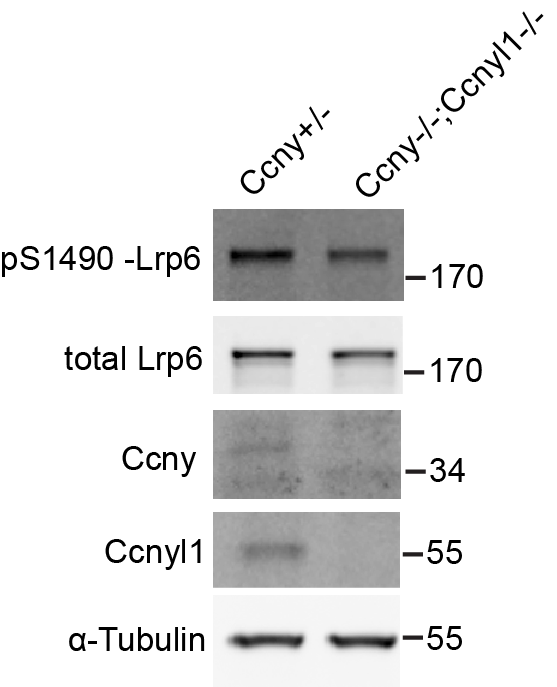

Supplement: S7 Fig — MEFs were isolated from Ccny heterozygous mutant and Ccnys DKO mouse embryos. Western analyses were performed to detect the indicated proteins. α-Tubulin served as loading control. (TIF) [file pgen.1006055.s007.tif]
